# Perspectives of scientists on disseminating research findings to non-research audiences

**DOI:** 10.1017/cts.2020.563

**Published:** 2020-12-07

**Authors:** Demetria M. McNeal, Russell E. Glasgow, Ross C. Brownson, Daniel D. Matlock, Pamela N. Peterson, Stacie L. Daugherty, Christopher E. Knoepke

**Affiliations:** 1 Department of Medicine, Division of General Internal Medicine, University of Colorado School of Medicine, Aurora, CO, USA; 2 Adult and Child Consortium of Outcome Research and Delivery Science (ACCORDS), University of Colorado School of Medicine, Aurora, CO, USA; 3 Department of Medicine, Department of Family Medicine, University of Colorado School of Medicine, Aurora, CO, USA; 4 VA Eastern Colorado Geriatric Research Education and Clinical Center, Denver, CO, USA; 5 Prevention Research Center in St. Louis, Brown School, Washington University in St. Louis, St. Louis, MO, USA; 6 Department of Surgery (Division of Public Health Sciences) and Siteman Cancer Center, Washington University School of Medicine, Washington University in St. Louis, St. Louis, MO, USA; 7 Department of Medicine, Division of Geriatrics, University of Colorado School of Medicine, Aurora, CO, USA; 8 Department of Medicine, Division of Cardiology, University of Colorado School of Medicine, Denver, CO, USA

**Keywords:** Dissemination, research practices, stakeholder engagement, qualitative survey

## Abstract

**Background:**

Little is known about practices used to disseminate findings to non-research, practitioner audiences. This study describes the perspectives, experience and activities of dissemination & implementation (D&I) scientists around disseminating their research findings.

**Methods:**

The study explored D&I scientists’ experiences and recommendations for assessment of dissemination activities to non-research audiences. Existing list serves were used to recruit scientists. Respondents were asked three open-ended questions on an Internet survey about dissemination activities, recommendations for changing evaluation systems and suggestions to improve their own dissemination of their work.

**Results:**

Surveys were completed by 159 scientists reporting some training, funding and/or publication history in D&I. Three themes emerged across each of the three open-ended questions. Question 1 on evaluation generated the themes of: 1a) promotional review; 1b) funding requirements and 1c) lack of acknowledgement of dissemination activities. Question 2 on recommended changes generated the themes of: 2a) dissemination as a requirement of the academic promotion process; 2b) requirement of dissemination plan and 2c) dissemination metrics. Question 3 on personal changes to improve dissemination generated the themes of: 3a) allocation of resources for dissemination activities; 3b) emerging dissemination channels and 3c) identify and address issues of priority for stakeholders.

**Conclusions:**

Our findings revealed different types of issues D&I scientists encounter when disseminating findings to clinical, public health or policy audiences and their suggestions to improve the process. Future research should consider key requirements which determine academic promotion and grant funding as an opportunity to expand dissemination efforts.

## Introduction

Timely translation of the benefits of health-related research is of international concern [1–3]. Both literature and policy assume that rapid translation of research into practice is ideal, yet delays in the operationalisation of research findings into real world practice persist [1]. While some lag is anticipated to ensure the safety and efficacy of new interventions or medical advances, we should aim to lessen the frequently cited 17-year lag time for 14% of evidence to reach practice [4,5]. Delays are seen as a waste of limited resources and an expense of potential patient benefit; however, research investigating factors which affect how scientist disseminate health research outside of the academic setting (e.g., to non-research clinical and policymaker audiences) is relatively scant [6].

Previous studies investigating the variety of dissemination practices to non-research audiences are limited. One study examined dissemination practices among public health scientists to identify factors related to dissemination efforts to public health officials. Study results found one-third of scientists rated their dissemination efforts as poor. However, many factors were significantly related to whether a researcher rated him/herself as excellent/good, including obligation to disseminate findings, importance of dissemination to his/her academic department and expectations by employer or by funder. Still, it is unclear how dissemination practices are integrated to annual performance metrics or funding mechanisms [7]. Further, prior work did not extensively assess perceived or existent requirements to disseminate findings to non-academic stakeholders. A greater understanding of these issues will illustrate the actual experience of scientists as they attempt to disseminate findings to non-research audiences while navigating an academic career and being responsive to requirements by grant funders.

A recent study by Knoepke et al. characterised current practices of dissemination to these audiences among dissemination and implementation (D&I) scientists [8]. More specifically, the study investigated potential characteristics associated with greater use of various dissemination strategies. While scientists were from diverse settings (e.g., clinical and community) and routinely engaged in a variety of dissemination-related activities, there were noticeable differences in the dissemination strategies used between scientists. These findings suggest additional factors related to academic setting or funding requirements may influence dissemination-related activities.

To further explore factors related to use of dissemination strategies meant to improve uptake of research findings in clinical and public practice among D&I scientists, the current project conducted an analysis of the qualitative responses of a previously-reported survey of D&I scientists [8]. The aims of this analysis are to: summarise D&I scientists’ experiences and opinions regarding dissemination activities’ importance to their work; identify how they are evaluated in this area of D&I science and summarise suggestions for how to improve the process to enhance translation to practice.

## Materials and Methods

### Sample and Procedures

Sample identification and online survey administration methods, as well as return characteristics of the dataset being analysed have been previously described [8]. In brief, we purposively sampled the opinions and insights of scientists with interest, training, funding and/or publication history in D&I. The survey asked respondents to report practices related to dissemination of findings to non-research audiences, as well as described methods by which they engage stakeholders in research to enhance translation. The project was approved by the Colorado Combined Institutional Review Board (COMIRB), including a waiver of written consent to participate.

### Data Collection

Surveys were distributed through Qualtrics® (when individual email addresses were available) or through electronic listservs as appropriate. Listserv distributions were conducted by managers of those listservs rather than by our study personnel due to confidentiality requirements. Potential scientists for whom we had individual email addresses received up to three reminder emails at 1-week intervals from April to May 2018. Responses were collected anonymously and respondents did not receive any incentive for participation.

The recent Knoepke et al. publication reports quantitative results of the survey regarding D&I scientists’ use of different dissemination strategies [8]. While the level of effort devoted to stakeholder engagement and non-academic dissemination appears to have increased over time, participants reported that the primary dissemination venues continued to be academic publications, conference presentations and reports to funders [7,8]. This difference persists despite a belief among D&I scientists that workshops, policy, briefs and face-to-face meetings are more effective channels for affecting practice or policy [8]. The focus of this paper is on the separate but related issue of responses to three open-ended questions: 1) how is your dissemination of research findings to non-research audiences evaluated?, 2) how would you improve the system for credit or recognition for disseminating research to non-research audiences? and 3) what is the one thing you could do that would most enhance your efforts to disseminate your research to non-research audiences?

### Data Analyses

The qualitative responses were transcribed verbatim into a word processing document. Qualitative responses were analysed by the primary authors (DMM, CEK), using a collaborative inductive open coding process for content analysis [9]. Both authors are PhD-trained researchers with experience in qualitative methods, health services research and D&I science. The analysis was conducted using inductive reasoning across three stages: 1) independent open coding followed by discussion of code descriptions to achieve consensus; 2) independent aggregation of codes into categories followed by discussion to reach consensus, development of category descriptions and identification of exemplars; 3) independent analysis followed by discussion to identify and describe overall themes, with categories across open-ended items examined for overlap, and to maintain objectivity. The project team reviewed and agreed upon key themes that emerged from the qualitative data. Thematic saturation was achieved as further observations and analysis revealed no new themes [10,11].

## Results

Demographic characteristics of surveyed scientists are presented in Table 1. Of the 159 scientist who completed the survey, all provided qualitative responses to at least one of the three open-response items. Specifically, we received 95 responses for questions 1 and 2 and 159 responses from question 3. The majority of scientists were from university or research settings in the USA (69%) or Canada (13%). They were from a mix of clinical (33%) and community settings (68%). The majority were from behavioural health (43%) or public health disciplines (42%); 26% had received formal training in D&I and there was a wide distribution in years since highest academic degree.

**Table 1.** Sample quotes from question 1: how is your dissemination of research findings to non-research audiences evaluated?

Themes	Sample quotes
Theme 1a: promotional review	“As part of promotion as a ‘service.’”
	“Based on reporting it in my annual faculty evaluation.”
	“Annual impact statement as part of annual review.”
Theme 1b: funding requirements	“It is part of the selection criteria used in the grant proposals that fund our work.”
	“By successful grants specifically for dissemination.”
	“Annual report of the research institute.”
Theme 1c: lack of acknowledgement of dissemination activities	“It is actually not really evaluated. It is sort of a side note in our giant dossiers.”
	“It is an important part of my job, but evaluated fairly subjectively.”

What follows are reports of key themes present in the data organised by each of the open-ended question. Additional illustrative quotes are also provided in Tables 1–3.

**Table 2.** Sample quotes from question 2: how would you improve the system for credit or recognition for disseminating research to non-research audiences?

Themes	Sample quotes
Theme 2a: dissemination as a requirement of the academic promotion process	“Make it more desirable to reach these audiences (more recognition within formal reports etc. – not everything should be based on publications).”
	“If it became a requirement, I think that would naturally result in some type of recognition in yearly evaluations, resulting in ‘credit’ of some type.”
	“Make it a separate item (rather than within service to the community) when evaluating career advancement (e.g., tenure, promotion, etc.).”
Theme 2b: requirement of dissemination plan	“Universities include in promotion, journals include in manuscripts, funders include in grant applications.”
	“Incorporate it into promotion and tenure guidelines for academic appointments and in consideration for VA promotion to GS14 status for investigators and increase amount of grant funding to pay for this.”
Theme 2c: dissemination metrics	“Having a clear definition of what dissemination to such an audience entails, and accordingly using it by the party giving that credit. The answer is hard to give as it also depends on who is giving the credit.”
	“First, you have to crack the measurement nut. In the same way that the coin of the research dissemination realm is number of publications, citations and journal impact factor, some equivalent, reliable and valid assessment of impact needs to be developed. Assuming such a metric exists or is developed then the next step needs to be working with the leadership to get them to see and accept the value of such an assessment, as well as the value of this performance dimension in the overall domain of performance for researchers.”

**Table 3.** Sample quotes from question 3: what is the one thing you could do that would most enhance your efforts to disseminate your research to non-research audiences?

Themes	Sample quotes
Theme 3a: allocation of resources for dissemination activities	“Better understanding of how to reach the audiences for scale-up.”
	“Have a dedicated staff person for communication and dissemination support.”
Theme 3b: emerging dissemination channels	“Visual abstracts for sharing on social media would likely garner more attention and interest – graphic design and visuals are very helpful communication tools.”
	“Produce lay summaries in patient facing research newsletters.”
Theme 3c: identify and address issues of priority for stakeholders	“(Establish) advisory board engaged from proposal to dissemination.”
	“Change the funding paradigm so it supports partnership and co-inquiry rather than single disease/time limited projects. Change the focus to context, and what the context needs now/next.”

### Question 1: How is Your Dissemination of Research Findings to Non-research Audiences Evaluated?

Of the 95 responses to this question, three distinct themes emerged from question 1: 1a) promotional review; 1b) funding requirements and 1c) lack of acknowledgement of dissemination activities.

#### 1a) Promotional review

Many respondents reported that when the dissemination of research findings to non-research audiences was evaluated, this evaluation occurred during an annual review. Researchers expressed a combination of ways in which the review took place. “When going up for promotion or other awards we indicate the impact our work has made on practice/policy. I also share events (i.e. policy changes) with my leadership through email.” It was also expressed that the evaluation of dissemination activities is considered under the “broad umbrella of community service.” Additionally, it was noted that evaluation of dissemination often occurred only “qualitatively” during the performance review conversation, perhaps indicating that these activities garnered less attention than more easily-quantifiable productivity.

#### 1b) Funding requirements

Overall, there was general agreement that many, but not all, grant funders expected dissemination of research findings. Researchers noted that “certain agencies specifically ask for this [dissemination] in an annual report” and also have “explicit expectations for our research to lead to clinical practice change.” Further, scientists mentioned specific funder requirements regarding dissemination expectations. “There is an expectation that evidence-based information and programmes will be taken to the community. Number of community contacts is part of what is measured, as well as support activities of these programs in community.” Scientists reported that particular funding agencies require “completion of milestones, presentation of results at meetings of various kinds” as well as the “reporting of how stakeholders (including patients, caregivers, policymakers, etc.) were engaged throughout the research process as means of assuring the dissemination of research.”

#### 1c) Lack of acknowledgement of dissemination activities

External evaluation of dissemination activities was often non-existent. A majority of researchers said that it is simply “not evaluated,” as demonstrated by such statements as “it is not an important part of my job.” Other respondents also noted they were either “unsure” or “not certain” if the university requires such an evaluation. “My institution only recognises peer-reviewed research in scholarly journals; dissemination among practitioners or stakeholders is derivative by an order of magnitude.” There were a modest number of scientists that stated the information was recorded in the “CV [curriculum vitae] section on dissemination” as well as through “publications, citations, media, social media etc.” Table 1 presents additional quotes of scientists’ experience with disseminating findings to non-research audiences.

### Question 2: How Would You Improve the System for Credit or Recognition for Disseminating Research to Non-research Audiences?

Of the 95 responses to this question, three distinct themes emerged from question 2: 2a) dissemination as a requirement of the academic promotion process; 2b) requirement of dissemination plan and 2c) dissemination metrics.

#### 2a) Dissemination as a requirement of the academic promotion process

Scientists described the need for a clear path for promotion that is inclusive of metrics associated with disseminating research, noting to “make it more desirable to reach these audiences (more recognition within formal reports etc. – not everything should be based on publications).” Specific suggestions provided by researchers were to “include it [dissemination] in metrics for academic promotion,” “recognition of practice-based reports equal to academic journals” and “link to tenure process.”

#### 2b) Requirement of dissemination plan

Scientists agreed in general that recognition for dissemination activities would receive more attention if they were a required element of grant proposals, noting to “add a dissemination outcome or plan in grant proposals; or a requirement to report dissemination channels and impact to non-research audiences …” Even more definitively, there were specific requests for “funders require it” and that “NIH put more weight on it.” Researchers more explicitly recommended to “make it [dissemination activities] a valued component of KT [knowledge translation] plans on grant applications, include a section in manuscripts for other ways to learn about the results of this study, assign higher priority to these activities in Canadian Common CV/salary awards.”

#### 2c) Dissemination metrics

Scientists generally believe that a clear measure to capture dissemination efforts will create a path for recognition within the academic setting. There was a general consensus that the current system lacks metrics in place to evaluate dissemination, noting the need to “develop a measure for how much time and effort a researcher puts into this, what the outcomes are, and make it as important as publishing in peer-reviewed journals.” Table 2 presents additional quotes of scientists’ recommendations on ways to improve the system.

### Question 3: What is the One Thing You Could do that Would Most Enhance Your Efforts to Disseminate Your Research to Non-research Audiences?

Of the 159 responses, three primary themes emerged from question 3: 3a) development of skills and allocation of resources for dissemination activities; 3b) emerging dissemination channels and 3c) identify and address issues of priority for stakeholders.

#### 3a) Development of skills and allocation of resources for dissemination activities

Scientists felt ill-equipped to effectively disseminate research findings. Specifically, it was noted that lack of financial resources and staffing to support such efforts are strong barriers to dissemination. Scientists stated explicitly, the need to “learn dissemination skills. Have a dedicated staff to assist with effective dissemination strategies. It is time-consuming and not all scientists are good at this part, so it would be good to work with creative staff and partners on this.” Scientists largely agreed that dissemination needs to begin early in the study design process, but is actually accomplished post-hoc, if it is even considered at all. Furthermore, scientists emphasised that dissemination and traditional research activities often require different skills and hiring staff specifically to support dissemination activities would improve impact. To rectify this, it was noted to prospectively prioritise dissemination activities, for example, including additional staffing and resources in research budgets. Further, the need for resources was continually emphasised.

#### 3b) Emerging dissemination channels

Scientists conveyed the need to increase their own use of emerging dissemination channels of reporting research findings. It was suggested to “create a website with regular blogs, social media posts and press releases” to share research findings to the non-research community. It was also recommended to create “visual abstracts for sharing on social media would likely garner more attention and interest – graphic design and visuals are very helpful communication tools.”

#### 3c) Identify and address issues of priority for stakeholders

Scientists referenced both practical and logistical challenges to meeting with stakeholders and the need to partner with them during all phases of the research process, noted that “non-researchers often do not care about or want to use research, so knowing how to make them interested in it to begin with would be my silver bullet.”

Specifically, the need to “better understand their [stakeholders] priorities” was expressed as well as the need to “to develop a formal dissemination plan as part of the research plan, including stakeholders in this process.” Table 3 presents additional quotes regarding researchers’ recommendations on ways to improve the system.

## Discussion

This study provides insights into how D&I scientists work is assessed in the academic setting with regard to dissemination to non-research audiences (e.g., practitioners, policymakers). Along with the recently published companion paper that reported frequency of use of different dissemination strategies, this report identified important considerations, needs, options and alternatives for both scientists and funding agencies [8]. D&I scientists overwhelmingly stated that methods of professional evaluation are lacking, citing an absence of recognition of dissemination activities as well as lack of resources for necessary support. In addition, scientists shared their beliefs of what might enhance the dissemination process.

D&I scientists reported a lack of acknowledgement or perceived importance of dissemination activities (outside of publications and scientific presentations) in annual or promotional evaluations. Scientists emphasised the need for funders to require dissemination activities as part of funding announcements and the research process, which could conceivably lead to greater emphasis on these activities in both proposals and funded projects. Finally, many scientists called for the creation and promotion of quantifiable metrics which could be applied to evaluating the impact of dissemination activities in the real-world.

The findings of this study fit into similar contexts of other studies that have explored research perspectives on dissemination. Tabak et al. conducted a cross-sectional study of 266 public health researchers at universities, the National Institutes of Health (NIH), and Centers for Disease Control and Prevention (CDC) [12]. The study authors compared self-rated effort to disseminate findings to non-research audiences across predictor variables in three categories: perceptions or reasons to disseminate, perceived expectation by employer/funders and professional training and experience. Results found one-third of scientists rated their dissemination efforts as poor. Many factors were significantly related to whether a researcher rated him/herself as excellent/good, including obligation to disseminate findings, whether dissemination was important to their academic department and expectations by grant funders. Prior studies have also found lack of internal support and expectations by funders [13], as contributing factors to marginal dissemination efforts. However, it is important to note that funders, for example, the Veteran’s Administration and the Patient-Centered Outcomes Research Center (PCORI) have been requiring a dissemination plan in grant proposals for years.

For scientists, the inclusions of dissemination activities as an academic measure are an important step that fulfills the requirement of performing “scholarship” [14]. Sharing research findings with the non-research community is an essential step to decreasing the 17-year gap that exists in translational science. The experiences provided indicate a growing awareness of and value for stakeholder engagement, but few suggestions were made regarding specifically how to pragmatically and effectively engage stakeholders throughout the research process. Many scientists noted barriers to working with stakeholders and some proposed the importance of understanding stakeholder priorities and including them in the research process from inception as a means of improving dissemination efforts.

The use of a variety of dissemination practices to reach non-research audiences (e.g., publication, meetings, webinars) has previously been described by Brownson et al. [7] One challenge to dissemination in clinical settings is competing priorities among stakeholders. For scientists, the priority is often on discovery, rather than application of new knowledge which is frequently reflected in academic promotional standards. By comparison, practitioners and policymakers often prioritise practical ways for applying discoveries for their respective settings [15,16]. This misalignment of priorities persists, as scientists recently reported their primary role as identifying effective interventions, not disseminating findings, particularly to non-research stakeholders [8]. The current study highlights the misalignment of priorities as it seems researchers prioritise promotional requirements while the academic structure establishes guidelines that may not necessarily consider dissemination of research.

Related to the theme of dissemination metrics, it is important to note that they do exist. In fact, current perspectives are encouraging the review of these fields for academic promotion and tenure [17]. Social media has become a critical space for the dissemination of knowledge and outreach to community and policymakers and also for the creation of communities of practice. For example, Altmetric (alternative metrics) have become one of the most commonly utilised metrics to track the impact of research articles across electronic and social media platforms [18]. Altmetrics are metrics and qualitative data that are complementary to traditional, citation-based metrics by estimating how many people have been exposed to and engaged with a deliverable through nontraditional channels (e.g., news, Twitter).These are intended to understand where and how a piece of research is being discussed and shared, both among other scholars, key stakeholders and in the general public, and can signal that research is changing a field of study or having any other number of tangible effects upon larger society (e.g., inclusion in a policy document). These metrics would theoretically help operationalise and streamline the discussion of dissemination activities during employment and promotion evaluation, incentivising researchers to devote resources to dissemination.

While these dissemination opportunities do exist, such efforts are not widespread and consistent, likely due to multiple challenges. For example, the appraisal of the quality and appropriateness of the content, the evaluation of impact on the academe and general populations, coupled with the creation of a system to reward scholars engaged in non-research dissemination [18], are all likely to play some role.

Further, in the case of clinical trials, the SPIRIT guidelines require a dissemination policy in the research protocol [19]. Specifically, it asks for a plan for investigators and sponsor to communicate trial results to participants, healthcare professionals, the public and other relevant groups (e.g., via publication, reporting in results databases or other data sharing arrangements). While the scientists surveyed in this study may not conduct clinical trials, it is important to note that SPIRIT requires dissemination as part of the protocol and can be expanded and modified for the academic community. The addition of the implications of the findings for modifications of this widely adapted tool would enhance the relevance to a wider framework of clinical and translational science.

Equally important to the discussion of study results is the acknowledgement of responses not found. Surprisingly, none of the scientist mentioned the importance of using theory to guide dissemination and implementation efforts. It is noteworthy that scientists did not mention this as part of what is needed to further disseminate their work, considering “theories guide implementation, facilitate the identification of determinants of implementation, guide the selection of implementation strategies, and inform all phases of research by helping to frame study questions and motivate hypotheses, anchor background literature, clarify constructs to be measured, depict relationships to be tested and contextualise results (pg.2)” [20].

In recent years, novel disciplines such as quality improvement, informatics and innovation have endeavoured to redefine the scope and nature of scholarly work in medical schools: however, other academic disciplines have lagged [21]. While current literature shows that many academic promotion and tenure committees in USA have adapted and modified their appraisal systems to reflect changes in the research environment, though it is not a common practice recognised across the greater academic milieu. Further research is needed to: a) identify ways to address the themes noted in this study, b) understand how to increase the priority for disseminating study findings to diverse audiences among D&I researchers, employers and funders and c) test the most effective ways to share results outside of academic settings.

## Limitations and Future Directions

Although informative, this study has several limitations. First, the survey was conducted online as opposed to the conduction of in-person interviews or focus groups, which reduced the opportunity to use prompts or to follow up with scientist for additional information. Second, the scientists sampled primarily specialise in public health, health services and D&I research, overlooking basic science researchers which may disseminate differently (e.g., through the patent process) and experience different academic and grant funding expectations.

Although a number of themes emerged from comments concerning needs, few specific innovative recommendations or examples emerged from researchers whose area of expertise is dissemination and implementation. Recommendations for future research and action might include case reports on the impact of requiring reporting of D&I activities in evaluation of performance and more specific requirements by funders and sharing and evaluation of different metrics of dissemination to non-research audiences.

## Conclusion

Bridging the persistent gap between scientific discovery and application to real-world policy and practice will require a host of structural changes to the appointment and promotions guidelines surrounding health research, as well as to the individual practices of D&I and performance evaluations [22]. In this project, we summarised perceived need for support from the perspective of D&I scientists, including the need to: 1) streamline the reporting and quantification of dissemination activities to non-research audiences for the purpose of employment evaluations, 2) more meaningful integration of dissemination planning into research at the design and proposal stage and 3) develop skills in dissemination activities outside of academic publications and presentations, including those more likely to reach audiences of practitioners and policymakers. Future inquiry and documentation of efforts to implement these recommendations should help reduce the research-translation gap.

## Availability of data and materials

The datasets used and/or analyzed during the current study are available on the Open Science Framework. More limited datasets are available under any reasonable data use agreement with the Principle Investigator (Knoepke).
